# Immune cell phenotype and functional defects in Netherton syndrome

**DOI:** 10.1186/s13023-018-0956-6

**Published:** 2018-11-26

**Authors:** Elina Eränkö, Mette Ilander, Mirja Tuomiranta, Antti Mäkitie, Tea Lassila, Anna Kreutzman, Paula Klemetti, Satu Mustjoki, Katariina Hannula-Jouppi, Annamari Ranki

**Affiliations:** 1 Department of Dermatology and Allergology, Helsinki University Hospital and University of Helsinki, P.O.Box 160, FI-00029 HUS, Helsinki, Finland; 2 Hematology Research Unit Helsinki, Department of Clinical Chemistry and Hematology, University of Helsinki and Helsinki University Hospital Comprehensive Cancer Center, P.O.Box 700, FI-00029 HUS, Helsinki, Finland; 3 Dermatology Unit, Seinäjoki Central Hospital, Hanneksenrinne 7, 60220 Seinäjoki, Finland; 4 Department of Otorhinolaryngology – Head and Neck Surgery, University of Helsinki and Helsinki University Hospital, P.O.Box 263, FI-00029 HUS, Helsinki, Finland; 5 Children’s hospital, Helsinki University Hospital, P.O.Box 28, FI-00029 HUS, Helsinki, Finland; 6 Folkhälsan Institute of Genetics, Helsinki, and Research Programs Unit, Molecular Neurology, University of Helsinki, Helsinki, Finland

**Keywords:** Netherton syndrome, T cell, B cell, NK cell, Cytotoxicity, Cytokine, Immunoglobulin therapy

## Abstract

**Background:**

Netherton syndrome (NS) is a rare life-threatening syndrome caused by *SPINK5* mutations leading to a skin barrier defect and a severe atopic diathesis. NS patients are prone to bacterial infections, but the understanding of the underlying immune deficiency is incomplete.

**Results:**

We analyzed blood lymphocyte phenotypes and function in relation to clinical infections in 11 Finnish NS patients, aged 3 to 17 years, and healthy age-matched controls. The proportion of B cells (CD19^+^) and naïve B cells (CD27^−^, IgD^+^) were high while memory B cells (CD27^+^) and switched memory B cells (CD27^+^IgM^−^IgD^−^), crucial for the secondary response to pathogens, was below or in the lowest quartile of the reference values in 8/11 (73%) and 9/11 (82%) patients, respectively. The proportion of activated non-differentiated B cells (CD21^low^, CD38l^ow^) was below or in the lowest quartile of the reference values in 10/11 (91%) patients. Despite normal T cell counts, the proportion of naïve CD4^+^ T cells was reduced significantly and the proportion of CD8^+^ T central memory significantly elevated. An increased proportion of CD57^+^ CD8^+^ T cells indicated increased differentiation potential of the T cells. The proportion of cytotoxic NK cells was elevated in NS patients in phenotypic analysis based on CD56DIM, CD16^+^ and CD27^−^ NK cells but in functional analysis, decreased expression of CD107a/b indicated impaired cytotoxicity.

The T and NK cell phenotype seen in NS patients also significantly differed from that of age-matched atopic dermatitis (AD) patients, indicating a distinctive profile in NS. The frequency of skin infections correlated with the proportion of CD62L^+^ T cells, naïve CD4^+^ and CD27^+^ CD8^+^ T cells and with activated B cells. Clinically beneficial intravenous immunoglobulin therapy (IVIG) increased naïve T cells and terminal differentiated effector memory CD8^+^ cells and decreased the proportion of activated B cells and plasmablasts in three patients studied.

**Conclusions:**

This study shows novel quantitative and functional aberrations in several lymphocyte subpopulations, which correlate with the frequency of infections in patients with Netherton syndrome. IVIG therapy normalized some dysbalancies and was clinically beneficial.

**Electronic supplementary material:**

The online version of this article (10.1186/s13023-018-0956-6) contains supplementary material, which is available to authorized users.

## Introduction

Netherton syndrome (NS, OMIM 266500) is a severe autosomal-recessive ichthyosiform genodermatosis with atopic manifestations, neonatal failure to thrive and recurrent skin infections. NS is caused by mutations in *SPINK5*, encoding an epidermal serine protease inhibitor LEKTI (lympho-epithelial Kazal-type-related inhibitor) [1]. The exact mechanism of the immunological dysfunction in NS has not yet been fully elucidated. NS patients have a selective antibody deficiency to bacterial polysaccharides [2], elevated serum IgE and IgG4 levels, low numbers of NK cells [3, 4] and increased levels of proinflammatory and Th17 pathway cytokines (IL-1β, IL-12, TNFα, IL-2, IL-19) both in serum and in skin [5, 6]. An early and broad allergic sensitization is typical [7].

In this study, we analyzed blood lymphocyte phenotypes and function in relation to clinical infections in a group of 11 Finnish NS patients and healthy age-matched controls.

## Patients and methods

### Patients

NS patients (*n* = 11, 3 female, 8 male), aged 3 to 17 years, were recruited from the Helsinki University Hospital and from the Seinäjoki Central Hospital, Finland. Cases V.1 and V.2, VI.1 and VI.2, and cases III.1, III.2 and III.3 are siblings. Six age-matched healthy controls were recruited among the elective surgery patients at the Children’s Hospital, Helsinki University Hospital with the exception of C1 (brother of II.2) and C2 (sister of IV.1).

Additionally, six age-matched children (1 female, 5 male), aged 2 to 7 years, were recruited from the Skin and Allergy Hospital, HUS, out-patient ward. All had a diagnosis of atopic dermatitis (AD) but no other diagnosed illnesses, symptoms or diagnosed allergies at the time of examination. One patient used desloratadine for itching occasionally, others did not have any oral medications. Four of the AD patients had used mild topical corticosteroids during the past month, two had used group II corticosteroids topically and one had used topical tacrolimus 0.03%.

All patients from the families I, II, III, IV and V have the same *SPINK5* mutation (c.652C > T (p.Arg218X)). Additional *SPINK5* mutations were found in the families VI (c.652C > T (p.Arg218X) and c.1220 + 1 G > C (IVS13 + 1 G > C)) and VIII (c.1048C > T p.(Arg350*) and c.2098G > T p.(Gly700*)**).** We previously reported that patients with the same mutation seem to have a similar clinical phenotype [7]. The samples were collected during the time period from August 2015 to May 2017 and additional AD patient samples in July 2018.

### Infection history

Data were collected from patient records of the Helsinki University Hospital and Seinäjoki Central Hospital, covering the time period from April 2003 to October 2017.

### IVIG treatment

Patients I.1, II.1 and VIII.1 received intravenous immunoglobulin (IVIG) therapy during the study period at a dose of 400 mg/kg/month. The protocol for II.1 was changed to weekly subcutaneous immunoglobulin administration (100 mg/kg) after five months of IVIG therapy. I.1 received IVIG for 11 months and VIII.1 for six months.

### Methods

Complete blood counts (CBC), analysis of lymphocyte subsets and serum immunoglobulin values were determined according to routine and accredited laboratory methods (http://www.huslab.fi). Mononuclear cells (MNCs) were isolated from peripheral blood by Ficoll gradient centrifugation (GE healthcare, Buckinghamshire, UK).

### Lymphocyte phenotyping

B cell subsets were determined according to routine methods (http://www.huslab.fi), and compared with pediatric reference values [8]. Populations were identified as followed: naïve cells (CD27^−^IgD^+^IgM^+^), memory cells (CD27^+^), non-switched cells (CD19^+^CD27^+^IgD^+^IgM^+^), switched cells (CD19^+^CD27^+^IgD^−^IgM^−^), activated cells (CD211^low^, CD38^low^) and transitional cells (CD38^++^IgM^++^). T cell phenotyping was performed with FACSAria II (BD Biosciences, San Diego, CA, USA) for CD45, CD3, CD4, CD45RA, CD62L, CD57 and CD27 surface markers and analyzed with FlowJo (Version 10.0,8r TreeStar) [9].

For NK cell phenotyping, CD45, CD3, CD14, CD19, CD56, CD16, CD57, CD62, CD27 and CD45RA markers were used as reported earlier (27). 50,000 CD45^+^ cells were acquired with FACSAria (BD Biosciences, San Diego, CA, USA) and analyzed with FlowJo (Version 10.0.8r, TreeStar) [9]. NK and T cell values and function were analyzed in comparison to age-matched healthy controls (see above). NK and T cell phenotypes were also analyzed in comparison to AD patients.

### Activation of T cells

To study the activation of T cells, MNCs were stimulated with anti-CD3, anti-CD28 and anti-CD49d [9].

### NK cell degranulation and cytokine secretion assays

To study the NK cell degranulation and cytokine secretion capacity, fresh MNCs were stimulated with K562, a CML cell line devoid of MHC class I expression [9]. Degranulation was measured by anti-CD107a-FITC and anti-CD107b-FITC and cytokine secretion by anti-IFNy and anti-TNFα and analyzed with FlowJo.

### LEKTI and AIRE expression in normal thymus and tonsillar tissue

Thymic tissue was obtained from pediatric patients undergoing thoracic surgery. Tonsillar tissue was obtained from 11 patients undergoing tonsillectomy due to either enlarged or chronically infected tonsils. All the patients and/or their parents gave written informed consent. All tissues were fixed in formalin and embedded in paraffin as routinely. LEKTI and AIRE immunostainings were performed on thymic and tonsillar tissue sections after heat-induced epitope retrieval in citrate buffer (pH 6.0, 10 min). Primary antibodies rabbit anti-LEKTI (1:100, HPA009067, Sigma-Aldrich, St- Louis, MO, USA) and mouse monoclonal anti-AIRE (1:100, clone 6.1) [10] were diluted in 1% BSA and applied on the sections for either 60 min at room temperature (anti-LEKTI) or overnight (anti-AIRE) at + 4 °C. The bound antibodies were visualized using Vector Universal ImmPress kit and Vector NovaRed (Vector Laboratories, Burlingame, CA) and counterstained with hematoxylin-eosin.

### Statistical analysis

Statistical analyses were conducted with SPSS Statistics 24 (SPSS Inc., an IBM company). Quantitative data were expressed as median values. Independent samples t test was used for comparison between two groups. Correlation between parameters was analyzed by Spearman’s rank correlation coefficient. All statistical analyses were performed with and SPSS Statistics 24 (SPSS Inc.). *P* < 0.05 was considered statistically significant.

## Results

### Lymphocyte phenotypes and subpopulations in NS patients

**Fig. 1:**
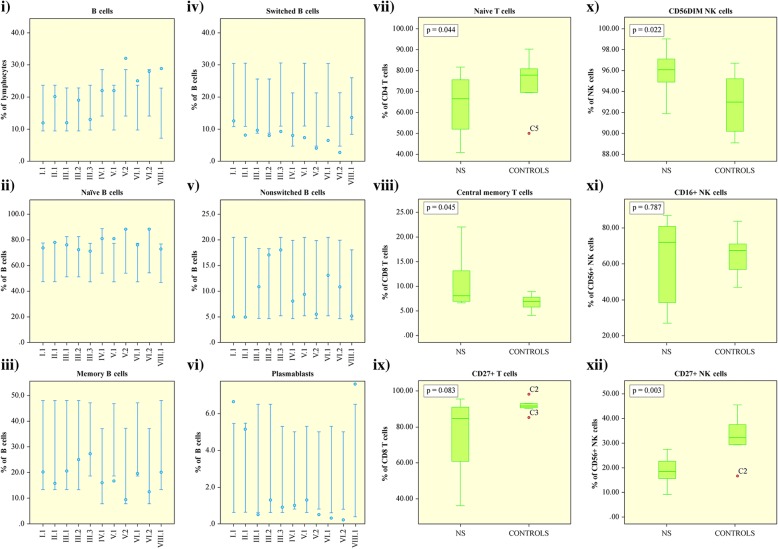
The most altered lymphocyte subtypes in Netherton syndrome. Relative proportion (%) of lymphocyte subtypes which differ in most patients with Netherton syndrome compared with age-matched healthy controls: (i) CD19^+^ B cells, (ii) naïve B cells, (iii) memory B cells, (iv) switched memory B cells, (v) non-switched memory B cells, (vi) plasmablasts, (vii) naïve CD4^+^ T cells, (viii) central memory CD8^+^ T cells, (ix) CD8^+^CD27^+^ T cells, (x) CD56^dim^ NK cells, (xi) CD56^+^CD16^+^ NK cells and (xii) CD56^+^CD27^+^ NK cells

**Fig. 2:**
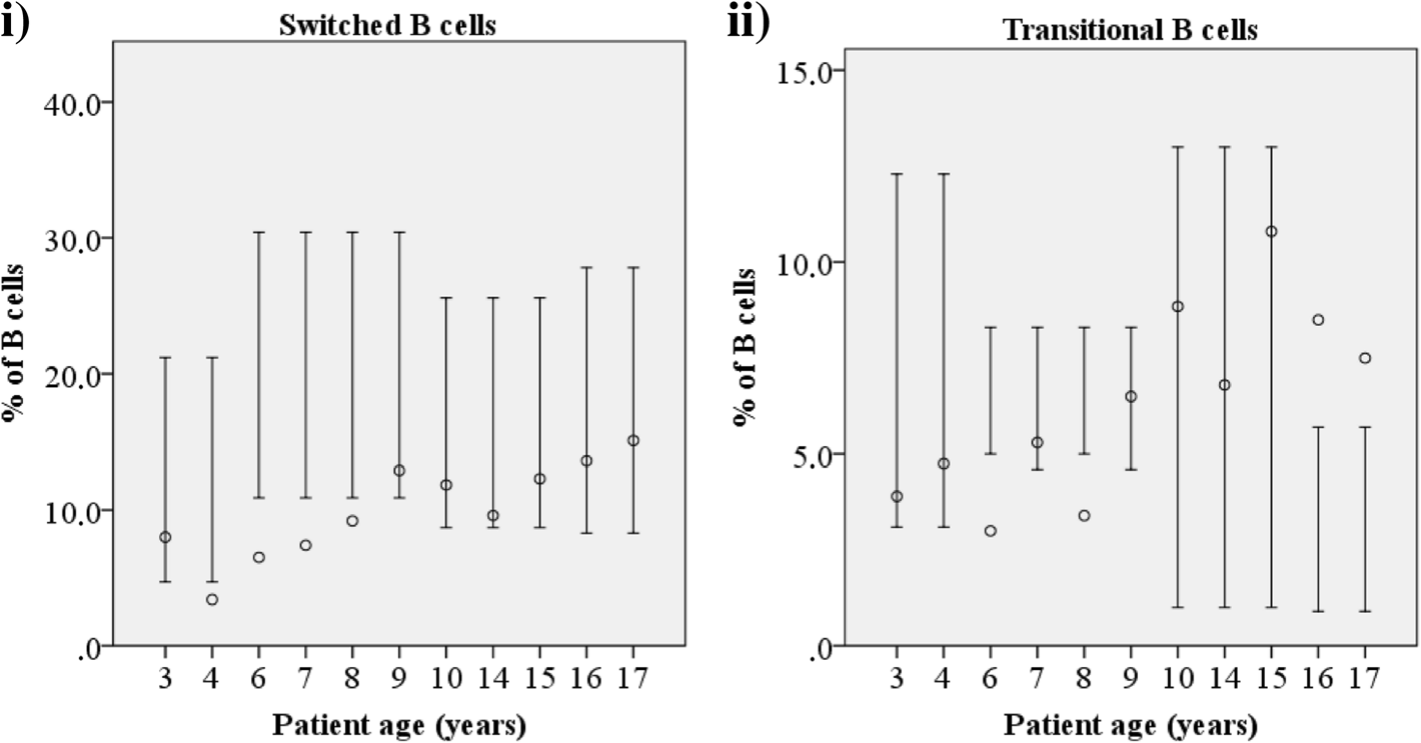
Proportions of switched and transitional B cells in different patient age groups. Altered proportion of switched (i) and transitional (ii) B cells are found in Netherton syndrome patients. The bars indicate the reference values for each age group and the circles indicate the mean of NS patients falling in the indicated age group. Repeated samples were taken from several patients at different ages

The complete blood counts and total T lymphocyte counts of NS patients were normal except for eosinophilia and occasional thrombocytosis. Serum immunoglobulin values were normal apart from elevated total-IgE, IgG4 and low IgG3 levels as we have reported earlier [3]. The proportion of CD19^+^ B cells was above the reference value in 3/11 (27%) patients and within the highest quartile of reference values in 4/11 (36%) (Fig. 1, i). The proportion of naïve B cells (CD27, IgD^+^) was within the highest quartile of reference values in 6/11 (55%) patients and above reference value in one patient (Fig. 1, ii). Memory B cells (CD27^+^) were within the lowest quartile of reference values in 7/11 (64%) patients and below the reference value in one (Fig. 1, iii). The proportion of switched memory B cells (SM, CD27^+^IgM^−^IgD^−^), which normally gradually increases up to 4 years of age and remains stable thereafter [11], was below the reference values in 7/11 (64%) and in the lowest quartile of reference values in two patients (Fig. 1, iv and Fig. 2, i). This B cell population is crucial for the secondary, T-dependent response to pathogens [12]. The proportion of non-switched memory B cells (NSM; CD27^+^IgM^+^IgD^+^), which normally increases during the first 5 years of life and is important for mucosal immunity [13], varied but was below reference values in 3/11 (27%) patients (Fig. 1, v). Pneumococcal vaccination responses were available for three patients (I.1, II.1 and VIII.1) and were interpreted as normal to most serotypes.

The proportion of activated non-differentiated B cells (CD21^low^, CD38l^ow^) was below reference values in 6/11 (55%) patients and within the lowest quartile in 4/11 (36%) patients while that of plasmablasts (CD38^++^, IgM^−^) was below the reference value in 5/11 (45%) patients (Fig. 1, vi), indicating defective secretion of immunoglobulins [14]. The proportion of transitional B cells (CD38^++^, IgM^++^) correlated with age: they were low in all patients below 8 years of age (Fig. 2, ii).

**Fig. 3:**
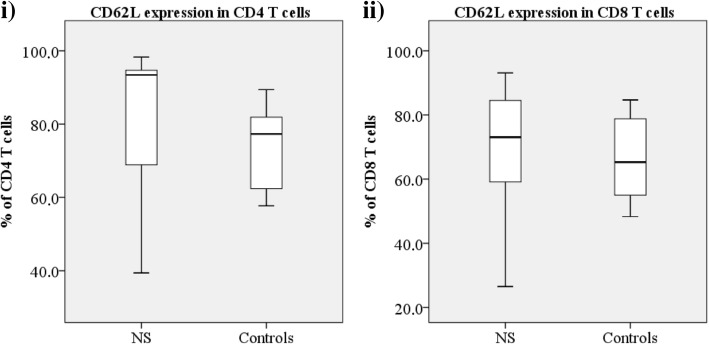
Elevated expression of CD62L in CD4^+^ and CD8^+^ T cells of NS patients and controls. Elevated CD62L expression in CD4^+^ (i, *p* = 0.460, patient median 93.4, control median 77.3) and in CD8^+^ (ii, *p* = 0.814, patient median 73.1, control median 65.3) T cells of NS patients and of age-matched controls

To investigate the characteristics of the T and NK cells, the cells were phenotyped with multicolor flow cytometry prior to and after stimulation (see Additional file 1). The proportion of naïve CD4^+^ T cells (CD45RA^+^CCR7^+^) was reduced significantly (*p* = 0.044, Fig. 1, vii) and the proportion of CD8^+^ T central memory (TCM) (Fig. 1, viii) was significantly elevated (*p* = 0.045). Two patients had an inverted CD4:CD8 T cell ratio. A significant increase in the proportion of CD57^+^ CD8^+^ T cells was observed (*p* = 0.037) and a slight decline in the proportion of CD27^+^CD8^+^ T cells (Fig. 1, ix). Also, the proportion of T cells (both CD4^+^ and CD8^+^) expressing the lymph node homing receptor CD62L/L-selectin was elevated in some patients (Fig. 3). As to the NK cells, the proportion of both CD56^dim^ and CD16^+^ cells, representing the more cytotoxic subset and also the majority of circulating NK cells [15, 16], was elevated in NS patients (*p* = 0.022 and *p* = 0.016, respectively; Fig. 1, x-xi). Also the proportion of CD27^+^ NK cells was significantly reduced (*p* = 0.003, Fig. 1, xii) suggesting enhanced cytotoxicity [17, 18].

Also when compared to age-matched AD patients, statistically significant alterations were seen in the T and NK cell phenotype (see Additional file 1 for details). In this comparison, NS patients had significantly more mature CD4^+^ and CD8^+^ T cells based on decreased proportion of naïve CD4^+^ cells and increased proportion of TCM, proportion of terminal differentiated effector memory (TEMRA) and CD57^+^ CD4^+^ T cells along with increased proportion of TCM CD8^+^ T cells. The impaired cytotoxicity and activation in NS patients were reflected as significantly decreased expression of CD27 in CD4^+^ cells also in this comparison. NS patients had also significantly more cytotoxic CD^56DIM^ and less CD56^BRIGHT^ NK cells than AD patients.

### Lymphocyte phenotype in family III

The three NS children of the family III showed more mature and cytotoxic T cells, compared with the other NS patients (Additional file 1) and had also an elevated proportion of CD57^+^ NK cells (*p* = 0.029) reflecting the maturity and high differentiation of the NK cells [19]. Controversially, the proportion of CD16+ NK cells was significantly decreased (*p* = 0.003), reflecting diminished cytotoxicity. The B cell profile was abnormal only in III.1.

### Functional capacity of peripheral blood T and NK cells in NS patients

**Fig. 4:**
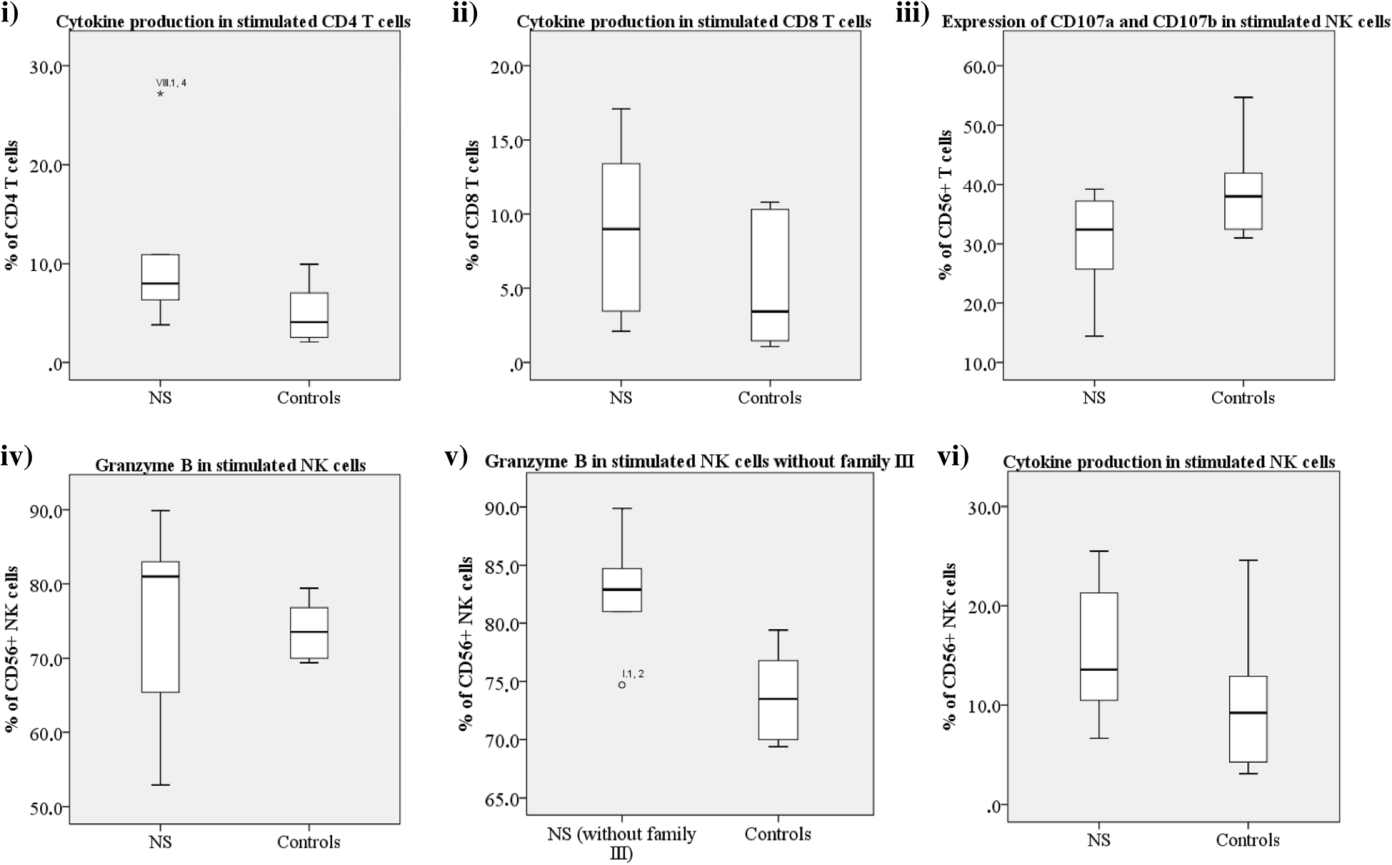
Functional aberrations in T and NK cells of NS patients. Increased cytokine production in stimulated T cells (i, *p* = 0.146, patient median 8.0, control median 4.1; and ii, *p* = 0.228, patient median 9.0, control median 3.4) and NK cells (vi, *p* = 0.199 patient median 13.6, control median 0.2), and decreased degranulation of NK cells (iii, *p* = 0.088, patient median 32.5, control median 38.0) despite high expression of granzyme B (iv, *p* = 0.903, patient median 81.0, control median 73.5 and v, *p* = 0.006, patient median without family III 82.9, control median 73.5) in stimulated NK cells of NS patients compared with healthy controls. Outliers in panels i and v are indicated with an asterisk and a circle, respectively

Upon stimulation of T cells, the production of both IFNγ and TNFα, by both CD4^+^ and CD8^+^ T cells, was increased compared with cells from matched healthy controls (Fig. 4, i-ii, Additional file 1). Despite elevated numbers of phenotypically cytotoxic NK cells, the expression of degranulation marker CD107a/b was significantly decreased in stimulated NK cells (Fig. 4., iii, Additional file 1) [20]. However, granzyme B expression was clearly increased in stimulated NK cells (CD56^dim^ and CD16^+^), excluding the family III patients in whom a significant decrease was observed instead (Fig. 4, iv-v and Additional file 1). Secretion of IFNγ and TNFα was increased in all NK cells indicating that cytokine secretion might compensate impaired functional cytotoxicity (Fig. 4, vi, Additional file 1).

### Association of infections and lymphocyte subpopulations

Typically, the NS children were hospitalized neonatally and most (8/10, 80%) received prolonged antibiotic therapy for skin infections (Table 1). No relevant viral or fungal infections were reported. The skin infection frequency and need for antibiotic usage correlated with the proportion of CD62L^+^ T cells, naïve CD4^+^ and CD27^+^ (intermediate memory) CD8 cells and with activated B cells (Table 2). An inverse correlation was found with memory B cells, NSM B cells, CD27^+^ (late mature) NK cells and with CD8^+^ TCM cells and CD57^+^ (terminally differentiated) T cells (Table 2). Minor correlations may have been missed because of the limited number of patients.

**Table 1:** Clinical infections and use of antibiotics in 11 Netherton syndrome patients

Family and patient	Age (years)	Antibiotic use in infancy ^i)^	Antibiotic prophylaxis ^ii)^	Bacterial skin infections ^iii)^	Conjunctivitis^iv)^	External otitis ^v)^	Life-time infection score ^vi)^
I.1	11	1	2	1	1	1	6
II.1	10	1	2	3	2	0	8
III.1	13	1	0	2	0	1	4
III.2	10	0	0	0	0	0	0
III.3	7	0	0	0	0	0	0
IV.1	3	1	1	0	0	1	3
V.1	7	1	1	1	0	0	3
V.2	4	1	2	1	0	1	5
VI.1	6	1	1	1	2	1	6
VI.2	3	1	2	1	0	1	5
VIII.1	17	1	1	1	0	1	4

i) 0 = no; 1 = yes

ii) 0 = no, 1 = yes, for 1–1.5 year period; 2 = yes, for > 1.5 years

iii) 0 = not more frequently than usual in the age group; 1 = recurrent infections at 1–1.5 years of age, 2 = recurrent infections at > 1.5 years of age, 3 = yes, for > 1.5 years, additionally recurrent *Staphylococcus aureus* (PLV) abscesses

iv) 0 = no; 1 = recurrent infections at 1–1.5 years of age; 2 = frequent conjunctivitis or constant need for antibiotic eye drops

v) 0 = not; 1 = frequent external otitis or constant need for topical antibiotics; case VIII.1 had recurrent otitis media

vi) Sum of the following scores: antibiotics in infancy, antibiotic prophylaxis, bacterial skin infections, conjunctivitis and external otitis. V.1 also suffered from sepsis after the neonatal period

**Table 2:** Specific lymphocyte populations associating with infection frequency in 11 Netherton syndrome patients as analyzed with Spearman’s rank-order correlation

Immunological parameter (%) ^i)^	Change in NS patients (*n* = 11) ^ii)^	Antibiotic prophylaxis (*r*_*s*_) ^iii)^	Antibiotic prophylaxis (*p* value)	Skin infection score ^iv)^ (*r*_*s*_*)*	Skin infections (p)	Total infection score ^iv)^ (*r*_*s*_*)*	Total infection score (p)
Positive correlation							
CD62L expression in CD4 T cells	increased	0.764	0.010	0.822	0.007	0.712	0.031
CD62L expression in CD8 T cells	increased	0.745	0.013	0.798	0.006	0.678	0.045
CD27 expression in CD8 T cells	decreased		> 0.05	0.71	0.014		> 0.05
Naïve CD4 T cells	decreased	0.742	0.009	0.798	0.006		> 0.05
Activated B cells	decreased		> 0.05	0.692	0.006	0.613	0.020
Negative correlation							
Memory B cells	decreased	−0.671	0.009		> 0.05		> 0.05
Non-switched B cells	decreased	−0.723	0.004	−0.698	0.006	−0.623	0.017
CD27 expression in NK cells	decreased	−0.788	0.004	−0.775	0.005		> 0.05
Central memory CD8 T cells	increased	−0.834	0.001	−0.71	0.014		> 0.05
CD57 expression in CD4 T cells	increased	−0.822	0.004	−0.798	0.006	−0.678	0.045
CD57 expression in CD8 T cells	increased	−0.564	0.070	−0.775	0.005		> 0.05

i) see precise definitions in Additional file 1

ii) compared with age-matched healthy persons

iii) Spearman’s rank correlation coefficient,

iv) Sum of the following scores as specified in Table 1: antibiotics in infancy (0–1), antibiotic prophylaxis (0–2), bacterial skin infections (0–3), conjunctivitis (0–2) and external otitis (0–1)

### Immunological changes during clinical improvement

**Fig. 5:**
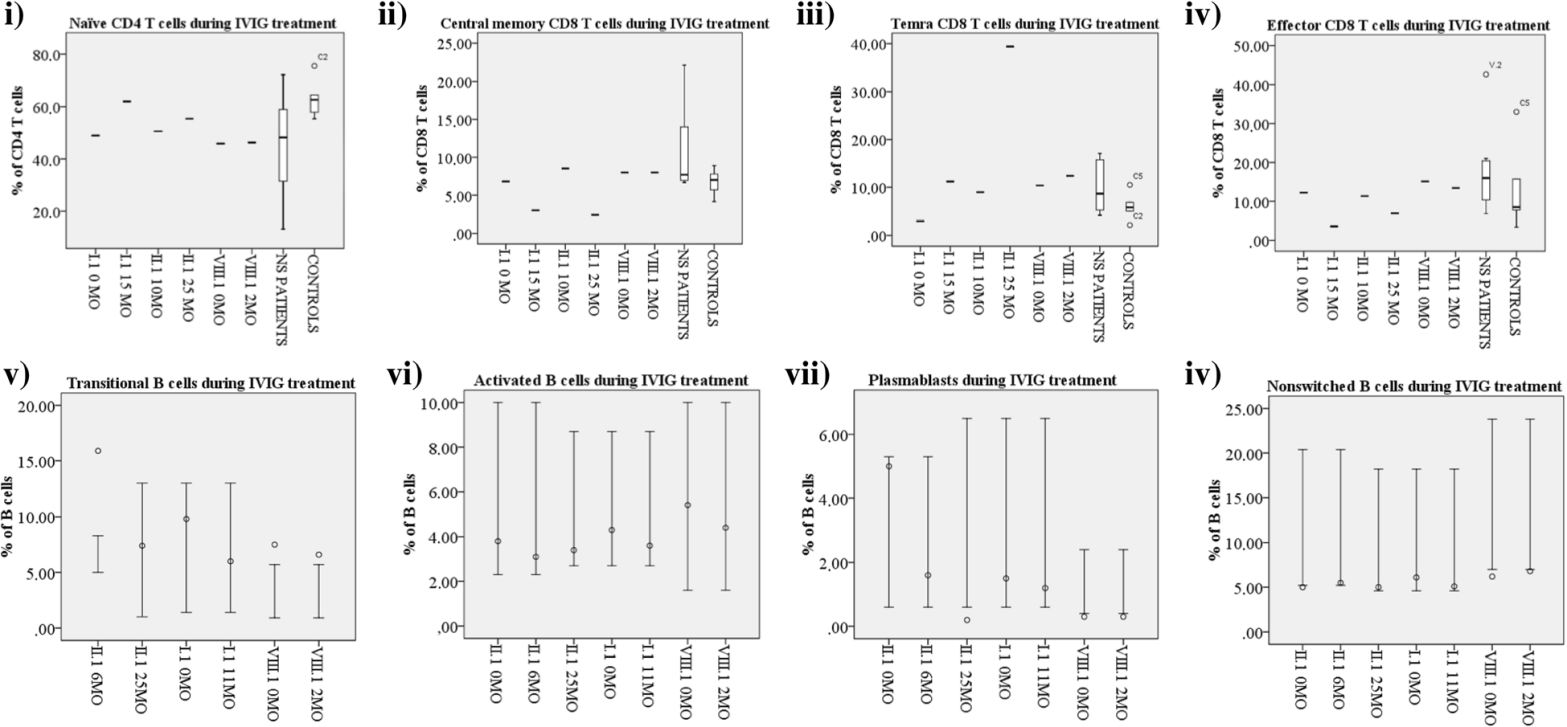
Changes in lymphocyte subclasses during IVIG treatment. Most relevant changes found in lymphocyte subclasses during IVIG treatment in NS patients. For B cell subclasses (v-viii) bars indicate the reference values for each age group and the circles indicate NS patient value falling in the specific age group

We had the opportunity to monitor the lymphocyte subclasses during IVIG therapy in three NS patients. IVIG has empirically proven beneficial in some NS cases [5], although the exact mechanism of action is not known. Patients I.1, II.1 and VIII.1 underwent IVIG therapy at the Helsinki University Hospital. S-IgG levels were normal in all and were thus not the target of the IVIG therapy. During therapy, the skin condition improved in I.1 and II.1 with less erythema, pruritus and flares and better tolerance to topical emollients. Importantly, no skin or other infections have occurred since and the daily emollient and topical corticosteroid use has decreased. After IVIG initiation, the proportion of naïve CD4 and CD8 cells increased and the proportion of TEM and TCM cells decreased (i.e. normalized; Fig. 5, i-iv and Additional file 1). The proportion of TEMRA CD8 cells rose in all three patients (Fig. 5, iii), while TEMRA CD4 cell values decreased (Additional file 1). No major changes in the proportions of B cells, memory B cells and SM B cells were observed (Additional file 1), while the proportions of transitional and activated B cells and plasmablasts decreased in all patients (Fig. 5, v-vii). Only minor changes in the NK cell phenotypes were observed (Additional file 1). The proportion of CD16^+^ cells increased further and CD27^+^ cells decreased considerably (studied in two patients) as expected [21], expanding the difference to the healthy controls.

Clinically, the hair of I.1 and II.1 has started to grow and thicken considerably and light microscopy revealed that they both now have predominantly normal hair and only infrequent trichorrhexis invaginata. For II.1 prior allergic symptoms to pollen and animals have subsided and a previously severe wheat allergy has subsided and tolerance to egg and nuts has also improved. Gastroesophageal reflux (GER) has also cleared off and medication has been terminated. However, severe cow’s milk allergy still prevails, and asthma symptoms have varied. For I.1 GER symptoms initially ceased but have relapsed to some extent. Only for patient VIII.1 (with a different mutation) the IVIG treatment was discontinued after six months due to lack of clear benefits. No IVIG-related significant pre- and post-treatment changes in total IgE levels or profiles on the ImmunoCAP ISAC microarray were seen in patients I.1 and II.1.

### LEKTI expression in relation to AIRE

**Fig. 6:**
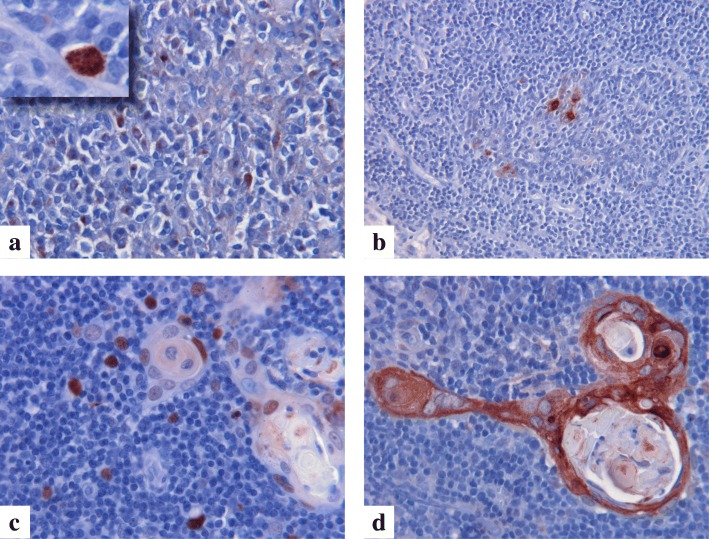
Expression of LEKTI and AIRE in lymphatic tissue. AIRE-expressing mTEC cells in tonsil (**a**), LEKTI-expressing cells in the corresponding tonsillar area (**b**), AIRE-expressing cells around Hassall’s corpuscles in the thymus (**c**) and LEKTI expression in a corresponding thymic area (**d**). The original magnification in panels is: (**a**) 20x, insert 100x, (**b**) 20x, (**c**) 40x and (**d**) 40x

The observed T cell imbalances in NS children may reflect a constant skin inflammation but may also be connected to defective LEKTI expression in medullary thymus, where maturing T cells are exposed to Autoimmune regulator (AIRE)-dependent self-antigen expression. Although thymic or tonsil samples from NS patients were not available, we looked for LEKTI expression in normal thymus and tonsil and found that LEKTI is expressed in the Hassall’s corpuscles in close proximity to AIRE-positive medullary thymic epithelial cells (mTEC) (Fig. 6). Interestingly, both AIRE and LEKTI seem to have a role in the end-stage mTEC differentiation. [22]

## Discussion

Here we characterize the aberrations of several B and T cell as well as NK cell subpopulations in a cohort of NS patients aged 3 to 17 years. Also, T and NK cell function was impaired although the patients did not suffer from viral infections.

Our observation of decreased SM B cells, important for the T cell dependent secondary response to pathogens is similar to previous findings by Renner et al. [5, 12]. In fact, the reduced proportion of SM B cells resembles that in common variable immune deficiency [23]. We also found a decrease in activated B cells and plasmablasts in NS patients, not previously reported, and the proportion of activated B cells positively correlated with the frequency of bacterial skin infections.

Within the T cell population, we observed a reduction in naïve T cells and a homeostatic expansion of effector memory cells. In healthy newborns, peripheral blood naïve T cell numbers are high and decline with increasing age while memory T cell numbers remain more constant. Pathogen-derived foreign antigens activate and induce naïve T cells to undergo massive expansion and to become effector cells [24]. The positive correlation between naïve T cells and infections may reflect the constant bacterial challenge on the skin.

The observed significant increase in the proportion of CD57^+^ CD8^+^ T cells, indicates increased differentiation of the T cells [19, 25, 26] and supports our previous hypothesis of more mature T cells in NS patients [3]. Importantly, the T and NK cell phenotype in NS patients differed from that observed in AD patients.

Interestingly, in addition to the skin, LEKTI is expressed in the Hassall’s corpuscles of thymus but LEKTI function in thymus is not known. We show that in normal human thymus, LEKTI is often in close proximity to AIRE-positive medullary thymic epithelial cells (mTEC) and based on this and previous data in mice [22] we anticipate LEKTI may have a role in the mTEC environment and may influence multiple aspects of intrathymic T cell maturation.

Also, our observation of a trend towards increased secretion of both IFNγ and TNFα by T and NK cells is new but is in line with the previously reported elevated serum TNFα values in NS patients [5]. Cytokine production might compensate the impaired NK cell functional cytotoxicity and activation, which was confirmed in this study in a larger patient cohort than previously reported [3, 5].

Interestingly, although the family III had the same Finnish founder mutation of *SPINK5* [3], they differed from other patients by having more mature T and NK cells and having their B cell phenotypes mostly in reference values. The quantity of infections in family III does not explain this difference since III.1 only suffered from frequent infections. It is still unclear what causes the difference between this and other families. Although we had the opportunity to monitor changes in the lymphocyte subclasses in only three NS patients under IVIG therapy, it was of interest to note that many of the aberrant cell populations changed towards the normal proportions along with clinical improvement. Notably, no skin infections were observed during the IVIG therapy.

## Conclusion

This study shows novel quantitative and functional aberrations in several lymphocyte subpopulations, which correlate with the frequency of infections in patients with Netherton syndrome.

## Additional file

Additional file 1: Lymphocyte phenotypes and populations in NS, AD and healthy control persons. The medians, *p* values and number of samples for all patient and control populations are given for each lymphocyte population (XLSX 28 kb)

## Abbreviations

AIRE: Autoimmune regulator
IFNγ: Interferon γ
IgE: Immunoglobulin E
IgG4: Immunoglobulin G4
IL-12: Interleukin-12
IL-1β: Interleukin-1β
IL-2: Interleukin-2
IVIG: Intra venous immunoglobulin
LEKTI: Lympho-epithelial Kazal-type-related inhibitor
mTEC: Medullary thymic epithelial cell
NK cells: Natural killer cells
NS: Netherton syndrome
NSM: Non-switched memory
OMIM: Online Mendelian Inheritance In Man
SM: Switched memory
SPINK5: Serine protease inhibitor of Kazal type 5
TCM: T central memory
TEM: Effector memory T cells
TEMRA: Terminal differentiated effector memory
TNFα: Tumor necrosis factor α
